# Volunteer responsibilities, motivations and challenges in implementation of the community-based health planning and services (CHPS) initiative in Ghana: qualitative evidence from two systems learning districts of the CHPS+ project

**DOI:** 10.1186/s12913-020-05348-6

**Published:** 2020-05-29

**Authors:** Margaret Kweku, Emmanuel Manu, Hubert Amu, Fortress Yayra Aku, Martin Adjuik, Elvis Enowbeyang Tarkang, Joyce Komesuor, Geoffery Adebayor Asalu, Norbert N. Amuna, Laud Ampomah Boateng, Justine Sefakor Alornyo, Roland Glover, Ayaga A. Bawah, Timothy Letsa, John Koku Awoonor-Williams, James F. Phillips, John Owusu Gyapong

**Affiliations:** 1 School of Public Health, University of Health and Allied Sciences, Hohoe, Ghana; 2 Volta Regional Health Directorate, Ghana Health Service, Ho, Ghana; 3 Regional Institute of Population Studies, University of Ghana, Legon, Accra, Ghana; 4 Policy Planning Monitoring and Evaluation Division, Ghana Health Service, Accra, Ghana; 5 Mailman School of Public Health, Columbia University, New York, USA; 6 Institute of Health Research, University of Health and Allied Sciences, Ho, Ghana

## Abstract

**Background:**

Community volunteerism is essential in the implementation of the Community-based Health Planning and Services (CHPS) in Ghana. We explored the responsibilities, motivations and challenges of community health management committees (CHMCs) in two CHPS+ Project districts in Ghana.

**Methods:**

We used a qualitative approach to collect data through 4 focus group discussions among a purposive sample of community health volunteers in December 2018 and analysed them thematically.

**Results:**

Community health management committees (CHMCs) were found to provide support in running the CHPS programme through resource mobilisation, monitoring of logistics, assisting the Community Health Officers (CHO) in the planning of CHPS activities, and the resolution of conflicts between CHOs and community members. The value, understanding and protective functions were the key motivations for serving on CHMCs. Financial, logistical and telecommunication challenges, lack of recognition and cooperation from community members, lack of motivation and lack of regular skill development training programmes for CHMC members who serve as traditional birth attendants (TBAs) were major challenges in CHMC volunteerism.

**Conclusion:**

Community health volunteerism needs to be prioritised by the Ghana Health Service and other health sector stakeholders to make it attractive for members to give off their best in the discharge of their responsibilities.

## Background

The 1978 International Conference on Primary Healthcare held in Almaty, Kazakhstan, expressed the need for urgent action by governments, health and development workers as well as the international community to protect and promote health for all across the globe [1]. It was also affirmed at the conference that the existing gross inequality in the health status of people, particularly between developed and developing countries as well as within countries was politically, socially, and economically unacceptable; hence, governments had to take the needed action to bridge the health inequality gap [1]. To achieve this, there is a need to make healthcare accessible to every citizen. The Ghana Health Service, therefore, implemented the Community-based Health Planning and Services (CHPS) as nationwide health policy in 2000 after its successful experimentation at Navrongo in the Northern Region of Ghana [2, 3].

Although primary healthcare in Ghana had been decentralized since independence from the national to the sub-district level prior the introduction of the CHPS, most communities in remote parts of the country had no access to basic healthcare services. Prior the introduction of CHPS, the structure of primary healthcare comprised of teaching hospitals at the top, followed by regional hospitals where curative services are delivered, with supervision and management support services offered by the Regional Health Administration [3]. At the district level, there are district hospitals with the District Health Administration playing a supervisory role and health centres at the sub-district level, offering both preventive and curative services. The health centres served as the first point of contact for healthcare services [4]. Only 65% of the population had immediate and easy access to healthcare at the hospitals while the remaining 35% had to cover a distance of between 1 and 9 miles in order to access healthcare, creating an unmet healthcare need in rural communities [4]. Hence, there was a need to further bring healthcare services closer to the doorsteps of rural communities, especially those in hard to reach areas of the country. As a result, the CHPS initiative was conceived to bridge the void by providing basic curative and preventive health services in underserved and hard to reach rural communities [5].

In CHPS implementation, several communities are put together, based on geographical proximity and called CHPS zones. A CHPS Compound, manned by one to three Community Health Officers (CHOs) serves as the health post which serves the zone in terms of healthcare delivery [6]. CHPS also depends on Community Health Volunteers, who support the CHOs with programme implementation [7]. The concept of community health volunteerism in the implementation of CHPS came from the realisation that communities can be active in the delivery of healthcare through the mobilisation of local resources to support health institutions in providing care as well as the design and mode of delivery of healthcare services [8]. It also emanates from the recognition that community health volunteers are key partners in health, particularly in villages across low and middle-income countries [9].

There are two sets of community volunteers in CHPS implementation. These are Community Health Volunteers and Community Health Management Committees (CHMCs) [8]. CHMCs are key health partners who play vital roles in the successful implementation of CHPS [7]. CHMCs, for instance, help in community participation and increase access to and utilisation of primary healthcare services through community mobilisation and education [9, 10]. Members are elected by the community to serve on the CHMC based on their willingness, acceptance and standing in society with the mandate to provide security for the CHO, settle disputes between the CHO and volunteers and community, promote the welfare of the CHO, supervise health volunteers and advocate for health [8]. Despite their immense contribution to healthcare delivery, community health volunteers often face several challenges, including logistical and financial challenges, as well as lack of support from community members [8].

### The CHPS+ project

The CHPS+ project is a scale-up of the Ghana Essential Health Interventions Programme (GEHIP) which was piloted in three administrative districts in the Upper East Region of Ghana from 2011 to 2016 to strengthen the implementation of CHPS [11]. The objective of GEHIP was to provide an integrated response to challenges that would strengthen district capabilities towards facilitation of CHPS scale-up. The scale-up, CHPS+ was initiated in 2017 and was expanded to include the Volta and Northern Regions [11].

CHPS+ is a collaboration project by the Ghana Health Service, the Mailman School of Public Health of the Columbia University, USA, the University of Ghana’s Regional Institute of Population Studies, Ghana, the University of Health and Allied Sciences’ School of Public Health, and the University for Development Studies with funding from the Doris Duke Charitable Foundation, USA. As part of the project implementation, the University of Health and Allied Sciences serves as training partner tasked with the primary purpose of building the capacity of the frontline health workers especially the CHOs through the development of short and long term training modules in the Volta Region of Ghana. The region was selected due to its high under-five mortality (61/1000) and fertility (4.8) rates in Ghana [12]. Two administrative districts (Nkwanta South Municipality and Central Tongu District) were selected by the Volta Regional Health Directorate of the Ghana Health Service and labelled as ‘Systems Learning Districts. This, therefore, influenced the choice of the two districts for the present study. The University of Health and Allied Sciences was chosen as a project partner based on its focus on problem-based learning approach (termed ‘vocational training programme’) adopted in training students upon its establishment in 2011 [13].

### Conceptual issues (the functional approach to volunteerism)

**Fig. 1:**
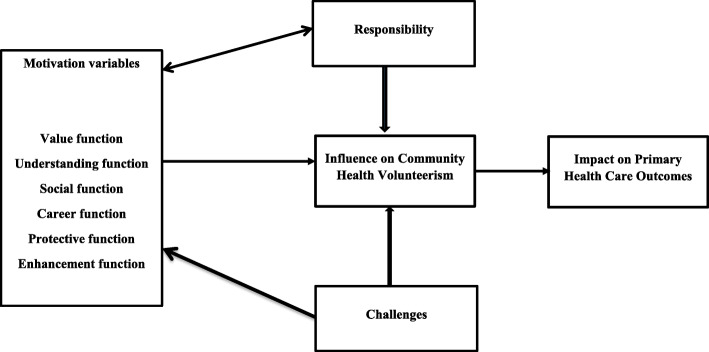
A functional approach to community health management volunteerism adapted from Clary et al. [14]

The concept of volunteerism and motivation has been explained under varied theories, including the functional approach to volunteerism [14] as shown in Fig. 1. The functional approach to volunteerism is based on the premise that volunteerism serves different functions for different people [15]. Thus, when volunteers’ essential motives for service are congruent to features of the environment that enable them to realise these motives, then volunteers will become more satisfied and are more likely to continue volunteering in future [14]. People often indulge in volunteerism to fulfil at least one of six functions; the value, the understanding, the career, the social, the protective and the esteem or enhancement. The value function deals with concerns for the welfare of others or society and contribution to society [14, 15]. Thus, the sole purpose of volunteerism is to help others. Some people also volunteer just because it will allow them to learn, practice and apply skills and abilities, known as the understanding function. Thirdly, some people volunteer as they see it as an avenue to boost their career prospects, thus the career function; where volunteerism is seen as an opportunity to acquire and develop new skills or establish contacts to boosts one’s chances of getting employed in future [16]. Moreover, others accept volunteer jobs due to strong normative or social pressure, known as the social function. This class of people agrees to do so because they want to get along with people in their reference group [14]. Regarding the protective function, people offer to volunteer to reduce the feeling of guilt for being more fortunate than others or to escape from one’s problems. Thus one feels guilty for not giving back to society. Lastly, some people offer to volunteer to enhance their self-esteem, self-confidence or to improve themselves, known as the enhancement function [14].

Considering the significance of the CHMC in the implementation of CHPS, there was the need to understand the responsibilities, motivations as well as the challenges that CHMCs in the two System Learning Districts encounter and how these impact on the conduct of their responsibilities based on the tenets of the functional approach to volunteerism. Such challenges could affect their motivation that could influence volunteerism. Addressing the challenges, therefore, could effectively motivate CHMC members to play their role in the implementation of the CHPS in Ghana.

## Methods

### Study setting

The study was conducted in the two CHPS+ Project Districts of the Volta Region of Ghana; Central Tongu District and Nkwanta South Municipality. The Volta Region is one of Ghana’s ten administrative regions with Ho as its capital. The region has 25 administrative districts/municipalities and a population of 2,118,252 with a growth rate of 2.5% as projected from the 2010 Population and Housing Census [17]. In terms of health facilities, the region has 514 health facilities; 271 CHPS compounds, 40 Clinics, hospitals, 156 health centres, 16 maternity homes, 2 polyclinics and 1 regional hospital [18].

The Central Tongu District has a total population of 71,331, a growth rate of 2.4% and the population covered by the CHPS-38,874 (54.5%). There is one district hospital, 4 health centres, 15 functional CHPS zones and 1 private maternity home. Mafi Zongo was selected as the Model zone for Central Tongu District. The rest, though were trained, are non-model zones according to Central Tongu District Health Directorate [19]**.** The Nkwanta South Municipality has a population of 140,121. There are 216 communities, 2 hospitals, 2 health centres and 21 functional CHPS zones. Salifu was selected as the Model zone for Nkwanta Municipality. The rest though were trained, they non-model zones according to Nkwanta South Municipal Health Directorate [20].

### Study design and participants

Focus Group Discussion (FGD) was the main qualitative methodological approach employed to observe the experiences of community volunteerism among CHMC members. FGDs have been described *as a thinking society in miniature* as it observes the co-construction of meaning and the development of knowledge at the broader group level [21, 22]. FGDs are often conceived as the society in its most basic form, as the interactions within the group allow researchers a controlled firsthand experience of how the social processes and makes sense of social phenomenon [22]. In this study, the focus groups consisted of people who knew each other and shared similar experiences in volunteerism. This was particularly useful as discussants contributed their unique encounters into a more complete picture of a social phenomenon, thus, volunteerism. This allowed the researchers to observe areas of strong emphasis, especially in aspects that discussants were most passionate about. Most importantly, the spontaneity of the collective reaction to a specific topic of interest gave the researchers an insight into the broader societal response [23].

Our core research team consisted of nine faculty members from the School of Public Health, five Ghana Health Service directors of health and three research consultants. The study participants comprised adults aged 18 years and above who were members of their respective CHMCs. Those who had lived in their respective communities for up to 6 months before the study were included. However, CHMC members who had travelled at the time of data collection or were seriously ill were excluded from the study. Purposive sampling technique was used to select the two System Learning Districts, the CHPS zones as well as the selection of individual participants for the study. Thus, all members of the CHMC from each of the two selected zones from each of the two System Learning Districts were selected. In all, 37 participants were recruited for the study.

### Interview guide

A semi-structured FGD guide (Appendix I), constructed in English and translated verbatim into Ewe and Akan languages, was used to conduct the interviews. The guide was developed by three research team members (MK, HA and EM). The guide was used to collect information on the socio-demographic characteristics of participants, their responsibilities and motivations for being CHMC members of their respective CHMCs as well as the challenges they faced in the carrying out their responsibilities.

### FGD interviews

The interviews were facilitated by six research assistants (all staff of the University of Health and Allied Sciences) and four supervisors who are faculty members constituting the core research team (MK, HA, EM and JK) from the same university. The research assistants were trained for 3 days on the data collection instrument and conceptualization of the CHPS+ project. This was done to ensure that all data collectors understood how to conduct the interviews to elicit appropriate responses from study participants. Pre-testing of the instrument was done at one of the CHPS zones in the Hohoe Municipality which is not a System Learning District. This was done to enable the research team to know how the participants understood and responded to the questions. After the pre-testing, the research team modified the questions and printed the final guide which was used for the study. Data collection began with an explanation of the purpose of the study to all participants. Participants were then organised into two focus groups per System Learning District, making a total of four. The first zone visited was coded Z1, the second Z2, the third Z3 and the fourth zone Z4. Responses were not linked to individuals to ensure anonymity and data collected was handled by only the research team to ensure confidentiality. In each focus group, alphabets were assigned to the participants and were used to identify them concerning their bio-demographic characteristics and contributions during the discussions. Each discussion lasted for about one and a half hours and was voice recorded with the permission of the participants. Handwritten notes were also taken during the interviews. After each interview, the facilitator and the PI made notes and discussed the preliminary observations. This preliminary analysis helped to improve the wording of the questions in subsequent interviews as well as in noting of areas that needed to be emphasized. Data collection was conducted in December 2018.

### Data analysis and data saturation

Audio recordings from each CHMC interview were transcribed verbatim from the Akan and Ewe languages to English by a certified language translator. The transcripts were then manually analysed using the thematic content analysis approach. Content analysis describes an analytic process that applies intuitive and interpretive approaches to systematically summarise textual data [24]. We thoroughly read through the various transcripts to gain a general sense of the information. Each transcript was put into segments and codes, which were descriptive, in terms of the subject matter in the transcript. Coding was done by writing the applicable codes in the margins of transcripts. The codes from the various transcripts were then compared and arranged under major topics. Sub-themes were then formed from the various topics by using the most expressive words for each set of codes. We then grouped related topics under various themes, based on already existing thematic areas reported in the literature designated as deductive codes as well as those that emerged from the data analysis process and were unique to this study, referred to as inductive codes [25]. Data saturation was deemed to have been achieved when there were no-emergence of new codes or themes from the transcripts.

### Trustworthiness of the study findings

The credibility of the study findings was ensured through the development of rapport between the research team and community and opinion leaders prior the study which opened participants up for interviewing without feeling hesitant to divulge sensitive but vital information. Dependability of the study findings was guaranteed through the input of professional qualitative researchers on the research team and from outside who studied and evaluated the study for perfection. Conformability was also ensured by seeing to it that participants went through the transcripts after to confirm that what we reported was what they said.

## Results

The results of the study are grouped under two headings. These are socio-demographic characteristics of participants and thematic results from the content analysis. Three broad thematic results emerged from the study; the roles and responsibilities of the CHMC, motivations for becoming a CHMC as well as the challenges facing CHMCs in the conduct of their duties. Quotes from the participants are used in presenting the data and also inform the structure of the discussion section.

### Socio-demographic characteristics of participants

A total of 37 participants from the two System Learning Districts (Nkwanta South and Central Tongu) were recruited for the study. Majority 16(43.2%) of the participants were aged 40–49 years and most 30(81.1%) were males. Majority 15(40.5%) completed second cycle institutions and most 34 (91.9%) were Christians. The dominant ethnic group were Ewe 20(54.1%) and most 36(97.3%) were married. Subsistence farming 27(73.0%) was the main occupation of participants. Less than half 18 (46.6%) of the participants were actively subscribed to the National Health Insurance Scheme.

### Responsibilities of the CHMC

Being a CHMC member comes with various responsibilities. Some of the responsibilities of the CHMC as identified by discussants were; provision of support for the CHPS facility and the CHO in terms of needs assessment conduction, overseeing the smooth running of the CHPS facility and mediating between the CHO and community members in disseminating information. They were also to be involved in the planning of activities of the CHO for the CHPS zone. Thirdly, as CHMC, they are supposed to be aware of the various activities undertaking by the CHO in their respective communities. Finally, they are to assist in resolving conflicts between the CHO and community members.

#### Provision of support services to the CHPS facility and the CHO

The CHMC provided services such as; mobilisation of community resources in building and maintaining CHPS compounds, home visiting to conduct a health needs assessment in the community, monitoring the wellbeing of the CHO and logistics availability and mobilising community members for health educational talks.

On mobilising resources for development and maintenance of respective CHPS compounds, discussants explained that they solicit for funds from community members in building their respective health facilities and nurses’ accommodation. They also organized women in the communities whenever the need be, to clean their respective health compounds to keep them tidy. The following quotes provide insight into the activities undertaking by the various CHMCs in that regard:

*“We [CHMC] mobilise our communities to help [physically, materially and financially] in putting up the building for the nurses” (CHMC member, Z1).*

*“We [CHMC] mobilise the women [in the community] to be clearing the place [of weeds] once in a while and all communities in the catchment area also come to clean up the place” (CHMC member, Z3).*

Regarding helping in conducting community health needs assessments, discussants explained that the CHMC regularly paid home visits in their respective communities to identify the pressing health needs of the people and provide the necessary guidance, support and feedback to the CHO, as mentioned by a CHMC member from zone 2:

*“Some of our responsibilities are that we visit communities to help fish out or identify sick persons. Also, when community members are sick, they come to us and we help them to the hospital” “when we see pregnant women, we ask whether they have gone for check-ups [or not]. If they answer ‘no’, we show them the way and the steps to the clinic. This is because some of the pregnant women are shy and reluctant to go to the hospital so we check up on them and send them to the CHO” (CHMC member, Z2).*

*“The nurse [CHO] cannot do everything by herself in all the communities. So we [CHMC volunteers] report cases and problems we find in our communities to the nurse during our home visits” (CHMC member, Z4).*

On their oversight responsibility, discussants explained that they played this by checking on the well-being and safety of the CHO and also monitor logistics availability at the facility to ensure that the limited resources they had were put into good use and also take the necessary action by reporting challenges to the district health directorate when the need be. A discussant narrated:

*“Sometimes, we [CHMC] group ourselves and visit the CHPS compound to check up on the [welfare] nurse. We find out about their wellbeing. We also find out if they have drugs [or not] then we send a report to the district director. We also have to see to it that the few medications we have at the clinic are not wasted by anybody” (CHMC member, Z1).*

*“These nurses are not from here, sacrificing to stay in this village to help us so as CHMC, it is our responsibility to ensure that they are safe” (CHMC member, Z4).*

The CHMC further assisted their respective CHOs in community mobilisation for health education activities by pre-informing community members of impending health education or promotion programmes. The CHMC also help in organising schools for health educational talks on behalf of their respective CHOs. According to the discussants, their involvement in community mobilisation enabled the CHO to undertake community and school health educational activities with ease. A discussant recounted:

*“We [CHMC] also help the nurses by disseminating information to community members. Also, if the nurse wants to organise [education] programmes, we first inform the community before the nurse goes ahead with the programme so that every community member can help or support the nurse” (CHMC member, Z2).*

*“We [CHMC] also inform the community not to go to their farms anytime the nurse [CHO] wants to come and talk to us about any health issue. Sometimes we even arrange with the schools to wait for the nurses to go and talk to them” (CHMC member, Z3).*

#### Assisting CHO with the planning of activities

Another salient responsibility of the CHMC was in assisting CHOs in the planning of activities for their various CHPS zones. Discussants noted that one of their core mandates was to be involved in the planning of activities for their respective zones. Hence, the CHOs were expected to be involving the committee in the planning their activities for their respective zones. This, they said was often the case as they often planned activities such as how to address challenges hampering the smooth running of CHPS compounds, drawing of action plans for the zones, strategies on treating of neglected tropical diseases, and on how to keep the CHPS compound clean. The following quotes sums discussants views:

*We [CHMC] also help with the planning of activities for our zone, even our action plan, the CHO, the committee and the chief and members of the community sat together to draw it* (CHMC member, Z3).

*… For instance, the recent injection against measles and rubella, he [CHO] informed us. What we do is that we have a communication centre, so if he [[CHO] wants to do any announcements, he [CHO] comes to us then we go on to inform the chief before making the announcement to community members. Also in the distribution of the oncho [onchocerciasis] medication that we just did, we decided on the date and time [for each community] together* (CHMC member, Z1).

#### Awareness of the services rendered by the CHO

Another key responsibility of the CHMC as mentioned by discussants was to be aware of the various services rendered by their respective CHOs in their communities. In this regard, discussants mentioned that they were fully aware of the activities undertaking by their respective CHOs as they were often involved in the planning of such activities. The following quotes explain participants’ views:

*“Yes, we [CHMC] are aware of what they [CHOs] do in our communities. The nurse sometimes visits [community members] from house to house to take care of the elderly who are sick and could not come to the hospital and they [CHOs] check blood pressures for those that are to not too sickly. The nurse also attends to children’s health during home visits” (CHMC member, Z1).*

*“Because some of us are not educated, when the nurse [CHO] tells us the prescription, we forget, so they [CHOs] are supposed to come to our homes to ensure we take the right dosage on time” (CHMC member, Z4).*

#### Conflict resolution

Conflict resolution between CHOs and community members was another key finding that emerged from the data. The CHMC was also responsible for resolving conflict situations. Discussants explained that conflicts often arise when there is disagreement overpayment for services rendered by a CHO to a community member, or over the quality of care [long waiting periods] received by community members. In some cases, conflicts arise between male CHOs and community members over sexual partners. In such situations, the CHMC is always on hand to quell tensions between a CHO and aggrieved community member to ensure peaceful coexistence. They explained:

*“One of our responsibilities is that whenever there is any confusion [misunderstanding] between a nurse and a community member [over payment for medication, child welfare clinic or during community visit], we [the CHMC] sit down with them [the aggrieved parties] to resolve the problem” (CHMC member, Z4).*

*“Sometimes there can be a problem between the nurse and a community member when the nurse takes too long to attend to them at the clinic. In such cases, we ensure that there is peace before everybody goes home. Sometimes too, the young male nurses [CHOs] and the town boys clash over girls and we [CHMC] have to intervene to make sure that there is peace” (CHMC member, Z2).*

### Motivations for becoming a CHMC member

Discussants provided various reasons as to why they accepted to work as CHMC members in their various CHPS zones. Notable reasons cited were the desire to provide leadership in their respective communities to improve health outcomes through the provision of effective leadership (the value function). Also, the opportunity for TBAs serving as CHMC members to upgrade their skills to better serve their communities better (the understanding function) motivated some participants to join their respective CHMCs. Lastly, affluent community members felt obliged to give back to their respective communities, which they felt could better be fulfilled through volunteerism (the protective function).

#### The value function as a motivation for volunteerism

Regarding the value function, some discussants explained that they wanted their communities to progress; hence they saw volunteerism as an opportunity to improve the health needs of their communities through the provision of good leadership to ensure an effective functional CHPS compound, as explained by a CHMC member from Z3; “*Every human institution needs people to lead them*”. The following quotes express the views of the participants:

*“We [CHMC members] accepted the offer because we wanted an improvement in our health needs and service delivery. Nobody from outside will come and make it [CHPS compound] work better for us except ourselves so it is our duty to serve [by leading] the committee to help improve our own health” (CHMC member, Z1).*

*“If the majority of the [community] members repose their trust in you, you do not need to reject it but accept it with happiness … … lead the community and help the clinic to deliver good services to the people, you don’t have to disappoint them. No one will come and develop this place [community] for us but ourselves”****(****CHMC member, Z3).*

#### *The understanding function* as motivation for volunteerism

Concerning the understanding function of volunteerism, some participants revealed that they voluntarily accepted to serve on their respective committees to get the opportunity to learn more skills to be better placed to serve their people in their respective roles. Discussants, specifically TBAs who were CHMC members, wanted a platform that could allow them to learn and apply their skills in the delivery of pregnant women in their respective communities. Below are excerpts from the discussants:

*“I am a traditional birth attendant so I thought that joining this committee [CHMC] will give me more opportunity to learn new skills and improve on my knowledge to be able to serve the community better. We [TBAs] were promised that we would be trained to be able to effectively perform our work so that motivated me a lot to join. The community is looking up to us [TBAs] so we can’t disappoint them*” (CHMC member, Z2).

*“Since we do not have a midwife in this community, we [TBAs] are the ones who often do deliveries in this community because, by the time you get a car to transport a woman to the hospital, it might be too late. So when I heard that they will be training some people on how to deliver babies, I saw an opportunity by joining this group [committee] to be able to learn such skills to help my people. It is very painful when a woman dies in labour so it is our [TBAs] duty to make sure that we deliver women safely” (CHMC member, Z2).*

#### The protective function as motivation for volunteerism

The protective function was also found to be a motivation for CHMC volunteerism. In this regard, affluent community members, per the socio-economic standards of each community, volunteered to serve on their respective CHMCs as they wanted to contribute their quota to help improve the health and socio-economic conditions in their respective communities. Thus, they felt obliged to give back to their communities, taking into consideration their good socio-economic standing in their communities as opposed to others. They, therefore, pledged to assist their respective CHOs in any way they could, through volunteerism, to improve the health conditions of their people. They explained:

*“I accepted the work because I think I am in a good position to help [the community] as compared to others [in the community]. If we the well-to-do ones in the community refuse to volunteer then who else will do it? Our nurse [CHO] is very hard-working so it will not be fair to refuse to assist him in his work, especially if you are in the position to do so. Per the community standards, some of us are economically better off so there is the need to help improve the condition [health] of others as well. It is the least some of us [the rich] can do” (CHMC member, Z1).*

*“In fact, I did not want to be part of this [CHMC] initially but I thought over it, together with my wife and decided that it is the right thing to do. If we the [somewhat] fortunate ones don’t do it, posterity will judge us [the affluent] should the community fail to get a good running health facility” (CHMC member, Z4).*

### Challenges faced by CHMCs

The data also revealed that CHMCs faces some challenges that affect their operations. The most common challenges faced by the CHMCs included financial challenges, logistical and technological challenges such as wellington boots, bicycles and poor telecommunication network, lack of recognition and cooperation from community members, lack of motivation for members and lack of refresher training courses for TBAs.

#### Financial challenges

Discussants explained that they were peasant farmers who do not get much income from their farming activities, hence the decision by the government for communities to solely finance CHPS compounds pose a challenge to the CHMC and the communities at large. They said:

*“We [community members] were here the last time when the district [health directorate] came and said that if we don’t provide security for the nurse, they are taking her away. We [community] know it is our duty to provide security put at least they [government] should help us in paying him since it is not easy to mobilise money every month to pay him. In such a case, it is us the committee members that suffer [by paying the security] because people [community members] think we [CHMC] are receiving some form of remuneration from the government”* (CHMC member, Z1).

*“Our security officer just vacated his post because we [the community] weren’t able to pay him for over five months so he got fed up with the job. We [the community] had to contribute some money to persuade him to come back to work since the nurse [CHO] was feeling unsafe being alone at the facility. This affects us [CHMC] a lot since we are also not paid for what we do. Some people don’t even want to be part of the committee again when our term ends. It is very demotivating” (CHMC member, Z3).*

#### Logistical and telecommunication challenges

The lack of logistics such as ‘Wellington boots’ bicycles, motorcycles for ease of movement and lack of mowing machines, hampered the activities of CHMC members and thus affected their output. Also, the non-availability of a telephone communication network in some communities served as a serious challenge in the performance of CHMC roles and responsibilities. Participants also lamented that the lack of good telecommunication network in their communities meant that they have to spend much time in organising themselves for meetings since they have to physically contact each other by going to their various homes instead of calling them on phones. This, they said, lowered the morale of CHMC members and affected their commitment towards work as some members were often not aware of meeting times and thus were not available for meetings. The following statement summarises their views.

*“We [the community] live in a muddy and swampy area. The formal director supplied us [CHMC] with wellington boots to help protect us. For example, when you are walking late in the night and you even step on a snake you won’t get bitten. That was the reason behind the former director giving us [CHMC] the Wellington boot and raincoat and some touch light to volunteers but for some time now, those things have not been coming again. Also, a mowing machine to be weeding the CHPS compound will also be a very good motivation but we [the community] have been weeding this place manually all this while. I am actually demotivated by this and I don’t know how long I can cope with this [being a CHMC member]. I am not sure how the government value us [the CHMC] but to me, it is clear that they don’t need us that much as we were told in the beginning so maybe its time to advice ourselves.*” *(CHMC member, Z4).*

*“Our [telecommunication] network here is not good so when you want to report a case to the nurse [CHO] or even call people for meetings, it becomes difficult. I will say that is one of the challenges I wished could be solved soon otherwise, some of us may not be members of this committee in future. Sometimes you [CHMC member] are on your way to the farm then they come and tell you that there is a meeting. If you [the CHMC member] knew of this earlier through a phone call, there will not have been the need to go in the first place. Our work is really affected because of this” (CHMC member, Z3).*

#### Lack of recognition and cooperation from the community

Another pressing challenge mentioned by the discussants was the lack of recognition and cooperation from community members. CHMC members felt that the voluntary work they were doing in their respective communities went unrecognized by their community members and thus affected their commitment and morale to work harder. Also, community members failed to cooperate with the CHMC in carrying out their duties. For instance, sometimes when some work had to be done at the CHPS compound, such as weeding or cleaning, community members failed to turn up. This is because community members feel that the CHMCs get remunerated by the government for their volunteerism and should, therefore, be solely responsible for all types of menial works at the CHPS compounds. Thus, the lack of recognition and cooperation from community members dampened the spirit of CHMCs to effectively give off their best.

Discussants’ views are summed below:

*“No one sees the importance or the sacrifice we [the CHMC] are making in this community. Some people even insult us [the CHMC members] on top of our sacrifice, without offering us anything, not even a thank you for all the good work we are doing. They [community members] think that we [the CHMC] are getting paid for what we do but that is not true. Look, it is yam farming season now and they are all gone to their farms but here we are talking to you, if at the end of the day we don’t get yams to feed our families they [the community] are the same people who will say we are lazy. We [CHMC] spend most of our time working for the people [community] but they are the same people that insult us. It is very sad” (CHMC member, Z3).*

*“One of the challenges we [the CHMC] have is that some community members are reluctant when we [CHMC] call for some communal work to be done. This is because they [community] feel that we are getting paid monthly by the government and for that matter, it is our job to do all the work at the clinic but that is not true. We get nothing for what we do and that actually make our work very difficult. Sometimes you are forced to ask yourself whether it is [volunteerism] worth it” (CHMC member, Z4).*

#### Lack of skills development training programmes

The lack of frequent skill development training programmes, at least for CHMC members who double as traditional birth attendants (TBAs), served as a disincentive to the CHMC and thus affected the work rate of such volunteers. Such CHMC members were frustrated that their skills have not been upgraded over the years and thus were not seen as important stakeholders in the CHPS initiative. Participants were therefore frustrated and demotivated to give off their best as CHMC members since they felt unvalued by health authorities. Participant from two zones explained:

*“We [the TBAs] used to go for routine training at Nkwanta [the district capital] but for close to 2 years now, we [TBAs] have not been invited for training. We [TBAs] need to update our skills in skilled delivery but the way they are treating us now, it seems we are no longer important to them. I think about it a lot and don’t know whether I should still be a member of this committee” (CHMC member, Z2).*

*“The only motivation I used to get was the training they used to organise for us [TBAs] but now there is nothing like that. I really don’t know what the problem with them [Health Directorate] is. Maybe it could be because they have sent a midwife to us [the community] now and no longer need our [TBAs] services but if that is the case then they should tell us [TBAs] to stop working” (CHMC member, Z 4).*

## Discussion

In this study, we investigated the responsibilities, motivations and challenges that CHMCs face in the implementation of the CHPS initiative in Ghana and how these challenges impact on volunteerism.

Concerning the responsibilities played by CHMCs, we intended to ascertain whether CHMCs were aware of their responsibilities in the implementation of CHPS in their various zones and whether they were carrying out those responsibilities. We found that the CHMC members were aware of their role(s) in the CHPS initiative and played four key responsibilities in their various zones: providing support to the CHO through resource mobilisation, home visiting, conducting a health needs assessment, monitoring logistics availability and ensuring the wellbeing of CHO, assisting the CHOs in the planning of CHPS activities, ensuring that they were aware of the activities undertaking by their respective CHOs; and helping in the amicable resolution of conflicts between the CHO and community members.

The responsibilities performed by the CHMCs in the System Learning Districts for the effective functioning of their respective CHPS compounds were in line with the requirements of the CHMC per the community health volunteers training manual developed by the [8]. Again, the roles and responsibilities of the CHMCs in the two System Learning Districts were similar to the roles of community health committees in some African countries such as Zimbabwe. In the case of Zimbabwe, the role of community health committees include; identification of priority health problems within communities, planning how to help communities raise their resources, organising and managing community input and advocating for the availability of resources for community health activities and inputs [26].

As an essential component of their responsibilities, discussants were always aware of the various services rendered by their respective CHOs. Home visits, educational talks (in communities and schools), disease control programmes, home deliveries, treatment of minor ailments and conduction of child welfare clinics (CWC) were some of the activities that were known to be conducted by CHOs in the various CHPs zones by the CHMCs. To the discussants, being aware of the various activities of the CHO did not only serve as an indicator of their performance but also served as a measure to keep their respective CHOs on their toes to deliver on their mandate. The finding was in line with the expectations of the CHMC, who are expected to perform an oversight responsibility [8]. Also, further studies support this finding [27].

We found that the CHMC was always involved in the planning and undertaking of health activities in their respective zones. Some of the activities the CHMC planned in unison with the CHOS include; how to run the health facility, development of action plan, planning on the treatment of neglected tropical diseases, and how to keep the CHPS compound tidy. Working together with people (teamwork) to improve the health status of the community is a skill that every CHO is supposed to acquire under the CHPS initiative [8]. As such, they are supposed to involve the CHMC in the planning of their activities. This element has been affirmed by research [28]. Hence, the involvement of the CHMCs in planning activities of the CHO is likely to improve the health outcomes of the communities. Even though discussants did not receive any form of recognition from their community members, they went ahead to carry out their duties to the best of their abilities. It could be argued that the opportunity to contribute to the improvement of individual and communal health outcomes through the strengthening of the CHPS initiative is a strong driving force to get community health committees to effectively do their work. Thus, the CHMCs in the two System Learning Districts were effectively carrying out their expected responsibilities in line with the training manual of the [2] despite a lack of recognition for volunteers.

Although CHMCs constitute an integral part of the successful implementation of the CHPS initiative, membership is purely voluntary. As such, one must have a strong motivation to serve on such a committee, taking into consideration the challenges that are associated with community health volunteerism. Three main reasons (provision of leadership to improve community health needs, opportunity to upgrade personal skills and the moral obligation of the affluent in society to give back to their communities), grouped into three thematic areas (the value function, the understanding function and the protective function respectively) under the functional approach of volunteerism [14] were the main reasons why members obliged to serve on their respective CHMCs. In other words, both intrinsic and extrinsic motivation [29] played a role in driving discussants to become community health volunteerism in our two System Learning Districts.

The value function was found to be an important contributor to volunteerism in the two System Learning Districts. Some CHMC members were of the view that they owed it to their communities to volunteer and provide the much-needed leadership to help properly steer the affairs of their respective CHPS facilities to maximise their performance and improve the health status of their communities thereby reducing the burden of existing health disparities in the country. This, they said, could be achieved through the conduction of proper community health needs assessment guided by effective leadership. Hence, some CHMC members believed that their presence on their respective CHMCs would enrich the performance of the committees to enable effective primary healthcare delivery in their zones by maximising the performance of their various CHPS compounds. Thus, they were concerned for the welfare of their communities and therefore decided to contribute to societal development. This assertion has been supported by studies conducted in the USA [30] and the United Kingdom [31].

The understanding function was also found to be another motivating variable for some CHMC members to volunteer on their respective CHMCs. Members were ready to work as volunteers to help improve the health outcomes of their respective communities by agreeing to serve on their respective CHMCs. CHMCs who were traditional birth attendants were of the view that being on their respective CHMC would enable them to improve on their knowledge on skilled delivery and thereby serve their communities better. To them, better understanding and skills in deliver will help improve delivery outcomes in their various communities even if they received no financial reward for the work they do. Studies conducted in Malaysia [32] and elsewhere in the world [33] reported similar findings where people volunteer to upgrade their skills. Furthermore, studies have found that while volunteers may not wish to receive some form of incentive for their work, most volunteers are more likely to give off their time when they perceive they are upgrading their knowledge, skills and understanding through volunteerism [34]. It could, therefore, be argued that volunteerism, it could lead to skills development becomes more attractive to prospective volunteers. The aspect of volunteerism in the CHPS initiative should, therefore, be made attractive by incorporating skills development training programmes for volunteers in their field of expertise to motivate them to give off their best.

Furthermore, the protective function, borne out of the feeling of guilt, motivated some discussants to serve on their various CHMCs. The financial and social standing of some community members pushed them to commit and work with their respective CHOs as a way of giving back to their communities. For such group of volunteers, they felt fortunate enough to have reached their current social standing and thus felt guilty to refuse to serve their people as volunteers to help improve communal health outcomes when they were better placed to do so. They, therefore, had a moral duty to play by giving back to their communities and found no better way than to volunteer to join their respective CHMCs. In short, they felt guilty for being socially and economically advantaged over other community members and had to do the needful by volunteering as CHMC members to help uplift their communities. This finding was supported by similar studies conducted elsewhere in the world [35, 36].

Concerning the challenges that CHMCs faced in the discharge of their duties, several issues were raised. These ranged from financial, logistical and telecommunication, lack recognition and support from community members, to lack of motivation and lack of frequent skill development training programmes for CHMCs who doubled as TBAs in their respective communities. These challenges were also found to have a negative influence on the performance of CHMCs as they were demotivated as a result. The Population Council and the Ghana Health Service [8] posited that community health volunteers, including CHMC members, are to be motivated by their respective communities to keep up with their voluntary work. Thus, they need constant encouragement, support and recognition from their communities. Hence, the lack of remuneration and support for community health volunteers, as found in this study, conflicts with such recommendation. Evidence suggests that projects that offer minimal economic incentives to community health volunteers tend to limit their focus and their performance while those that offer some form of incentives maximise the potential of such workers [37, 38]. The lack of motivation was therefore found to demotivate community health volunteers since they felt neglected. Efforts should, therefore, be made to find ways of remunerating community health volunteers to encourage them to give off their best if successful implementation and sustenance of the CHPS initiative is to be achieved.

Logistical and technological challenges such as the unreliable supply of proper working gear, lack of bicycles and tricycles as poor telecommunication network also served as a demotivation for some CHMC members and hampered their work. Studies have shown that lack of these supplies to volunteer health workers diminishes the effectiveness and seriousness they attach to their work [33, 39]. The lack of supply of basic logistics such as raincoat and bicycles has therefore led apathy and bitterness on the part of some CHMC members, thereby affecting their maximal cooperation and operations as community volunteers which is likely to impact on the running of their respective CHPS compounds for communal benefit. The lack of telecommunication services in some of the communities also hampered the work of CHMC members. As members are drawn from different communities and villages in the CHPS zone to form the CHMC, some members hail from communities where there is weak or no telecommunication signal at all, thus making telephonic calls difficult. This affected the organization and attendance of meetings of the CHMC. Moreover, valuable time is lost in situations where there is the need to report emergency cases to the CHO or when referrals need to be made as key stakeholders such as ambulance drivers, become unreachable due to poor telecommunication network. Participants were therefore handicapped and demoralized to effectively perform their duties as volunteers. Meanwhile, it has been found that to effectively mobilise communities for positive health outcomes, there is need for an effective communication network in these rural communities [40, 41], hence, efforts should be made to improve on logistical and telecommunication challenges facing communities under the various CHPS zones in Ghana.

Moreover, as part of the community health volunteers training manual, various suggestions were made as to the need to recognize and motivate community health volunteers by the Ghana Health Service. To this end, communities are expected to assist CHMC members on their farms or businesses, or provide them with foodstuffs as well as pay their transportation costs to training programmes and other meetings. Also, communities are expected to provide logistics such as working gear, bicycles and motorcycles as well as money to cover maintenance cost [8]. Unfortunately, we established that communities no longer offer these motivational and supportive services to community health volunteers, including the CHMC. The lack of recognition, therefore, served as a demotivation to CHMC volunteers and thus hampered their work.

The last challenge, as stipulated by discussants, especially those who also served as TBAs, was their disappointment in the lack of periodic refresher courses on their skills. The importance of training courses as a source of motivation, capacity building and performance for volunteers has been scientifically established [42]. Hence, failure to provide such an incentive which is of utmost importance to people who play a crucial role in communal delivery outcomes served as a demotivation to CHMCs, especially TBAs and thus, negatively affected their output as effective community health volunteers.

## Conclusion

The respective CHMCs performed their responsibilities in ensuring quality primary healthcare delivery in their communities. They helped in planning the activities of the CHO for the CHPS zone, conducted a community health needs assessment and with the development of action plans. They also served as liaison officers between the community and the CHO, in treatment of neglected tropical diseases, in cleaning the CHPS compound and also resolved conflicts between the CHO and the community. Three forms of motivation were found to influence volunteerism; the value function (desire to serve to improve the health needs of their respective communities), understanding function (skills learning) and guilt function (the need for the privileged to serve their communities). Although logistical and telecommunication challenges were the main reported, CHMCs also faced other challenges including financial challenge, lack of recognition and cooperation from community members, and lack of refresher training courses for CHMC members who are also TBAs.

Based on our findings, we conclude that although most CHMC members are intrinsically motivated to carry on with their responsibilities, the challenges they face demotivate them to give off their best. Consequently, this negatively impacts the performance of the CHPS zones and affects healthcare delivery outcomes in the communities. We, therefore, recommend that volunteerism under the CHPS initiative needs to be given more priority by the Ghana Health Service and other stakeholders in Ghana’s health industry than is currently done to ensure that challenges are addressed to make it more attractive for volunteers to give off their best. This will consequently influence other community members to serve as volunteers in the implementation of CHPS.

## Supplementary information

**Additional file 1: Appendix I.** Focus group discussion guide

## Data Availability

The datasets used and/or analysed during the current study available from the corresponding author on reasonable request.

## Abbreviations

CHMC: Community Health Management Committee
CHO: Community Health Officers
CHPS: Community-based Health Planning and Services
FGD: Focus Group Discussion
GEHIP: Ghana Essential Health Interventions Programme
TBAs: Traditional Birth Attendants
