# Supplementary material for: Volunteer responsibilities, motivations and challenges in implementation of the community-based health planning and services (CHPS) initiative in Ghana: qualitative evidence from two systems learning districts of the CHPS+ project
Source: BMC Health Serv Res. 2020 May 29;20:482. doi: 10.1186/s12913-020-05348-6 (PMC7260774; doi:10.1186/s12913-020-05348-6)
Supplement: Supplementary file 1 — Additional file 1: Appendix I. Focus group discussion guide [file 12913_2020_5348_MOESM1_ESM.docx]

**APPENDIX I: FOCUS GROUP DISCUSSION GUIDE**

**UNIVERSITY OF HEALTH AND ALLIED SCIENCES, HO, GHANA**

**SCHOOL OF PUBLIC HEALTH (CHPS+ PROJECT)**

FGD ID: ­­­­­­­­­­­­­­­_____

Participant ID: ____________________

Date of interview (DD/MM/YY): / _/_____

Time of interview: Start ___________End____________

District: Zone Name:

| **100: SOCIO-DEMOGRAPHIC CHARACTERISTICS**  Fill in or circle the appropriate responses for questions 101 to 110 | | |
| --- | --- | --- |
| No | Variable | Attributes |
| 101 | Sex | 1. Male……………………………………………..……1 2. Female ………………………………………….……2 |
| 102 | Age (In completed years) | ­­­­­­­­­­­­­­­­­­­­­­__________________ |
| 103 | Religion | 1. Christianity……………………...……..……...…..….1 2. Islam…………………………………..….........…..…2 3. African Traditional………………….…….............….3 4. Other (specify)………………………………… |
| 104 | Marital status? | 1. Married or living together……………..…………..…1 2. Divorced/Separated…………………..…….…….…..2 3. Widowed……………………………..……………....3 4. Never married and never lived together……………...4 |
| 105 | Ethnicity | 1. Ewe…………………………………………………...1 2. Akan……………………………………....………….2 3. Guan……………………………………..……….…..3 4. Ga-Dangme………………………………............…..4 5. Mole-Dagbani…………………………………….….5 6. Other (specify) ___________________________ |
| 106 | Highest educational level | 1. No formal education………………………………….1 2. Primary……………………………………………….2 3. Junior High School (JSS/JHS)………………………3 4. SHS/Vocational/A’level/O’level……………………4 5. Tertiary………………………………………………5 |
| 107 | Main occupation | __________________________ |
| 108 | Average monthly income | __________________________ |
| 109 | NHIS Status | 1. Active subscriber…………………………..……..1 2. Inactive subscriber ……………………………….2 3. Non-subscriber……………………………….…..3 |

**Introduction**

**Facilitator’s welcome, introduction and instructions to participants**

**Welcome** and thank you all for agreeing to take part in this discussion. You have been invited to participate in this discussion because you are members of the Community Health Management Committee (CHMC) and your views regarding CHPS implementation are important. Thank you for making time despite your busy schedules, we appreciate your time.

**Introduction:** This focus group discussion is designed to assess your general knowledge and contribution to CHPS implementation as CHMC members. Specifically, we want to know why you agreed to serve on the CHMC, the role you play as CHMC and the challenges that you face in carrying out these roles. The discussion will last for about an hour. We will be tape recording the discussion. This is to ensure that we have a good record of the information gathered.

**Anonymity:** Despite being taped, we will like to assure you that the discussion will be anonymous. To achieve this, we will be giving you letters (eg. A, B, C, D) which will replace your names. So, whenever you want to respond to an issue, you will only have to mention that letter. To ensure that information collected from the discussion is protected from unauthorized access, the tapes will be kept safely in a locked facility before they are transcribed and used for the purpose for which they are collected and destroyed after about a year.

You are required to comment as accurately and truthfully as possible. If there are any questions or discussions that you do not wish to answer or participate in, you do not have to do so; however please try to answer and be as candid as possible. If any of you is in contention/support with/of what another participant says, the person should just mention that particular participant’s pseudonym (letter). For example: I am participant “A” and I disagree/agree with what participant “B” said.

**Ground rules**

- The most important rule is that only one person speaks at a time. There may be a temptation to jump in when someone is talking but please wait until they have finished.
- There are no right or wrong answers.
- You do not have to speak in any particular order.
- When you do have something to say, please raise your hand and we will call you to speak.

Does anyone have any questions?

Can we please begin the discussion? (If yes, then start the main discussion)

**Introductory question**

- I am going to give you a couple of minutes to think about your experiences of being a member of the CHMC in relation to CHPS implementation. Is anyone ready to share his or her experiences?

**ROLE OF CHMC IN CHPS IMPLEMENTATION**

1. How did you become members of the CHMC?
2. What informed your decisions to serve as members of the CHMC? (Probe: financial gain, influence, support for community activities)
3. What are your responsibilities as the CHMC?
4. Have you been trained on your roles as a CHMC? (If yes, probe when the training was conducted and by whom?)
5. When did you have your last CHMC meeting? (probe for specific dates/approximate periods)
6. What issues were discussed at the last meeting?
7. What services do CHPS provide to community members? (Probe: Home visits, school health services, treatment of minor illnesses, defaulter tracing, reproductive health services, emergency delivery, health education, community health needs assessment etc.)
8. In what ways do you as CHMC support service delivery by the CHOs?
9. Do you have a CHAP? (Probe: If yes, when and how was the CHAP developed?

If no, why?)

1. **Have the CHOs been involving the CHMC in the planning of their activities? (Probe for the level of involvement and specific activities the CHMC has been involved in planning)**
2. Currently, what plan do you have in place for the maintenance of the CHPS compound? (Probe: Ascertain when the plan was developed and how it is being implemented. If no current plan is in place, what was the most recent plan and when was it developed as well as implemented)
3. What are the Community Emergency Transport Systems (CETS) that you have in place? (Probe: when it was developed and maintenance plan in place. If no CETS in place, why?)
4. What challenges do you face in rendering your duties as CHMC for the effective implementation of CHPS? (Probe: Lack of financial and technical support, negative attitude of CHOs/community, lack of our expertise)
5. What additional responsibilities do you think you can perform to help improve the implementation of CHPS in this zone?
6. Is there anything we have not discussed, but which you would like to share?

**CONCLUSION**

- Thank you for participating in this discussion. This has been a very successful discussion
- Your opinions will be a valuable asset to the study
- We hope you have found the discussion interesting
- If there is anything you are unhappy with or wish to complain about, please contact the local PI (provide contact of the Dean or Coordinator) or speak with any of us later
- I would like to remind you that any comments featuring in this report will be anonymous.
